# Large-Scale Transcriptomics-Driven Approach Revealed Overexpression of *CRNDE* as a Poor Survival Prognosis Biomarker in Glioblastoma

**DOI:** 10.3390/cancers13143419

**Published:** 2021-07-08

**Authors:** Maxim Sorokin, Mikhail Raevskiy, Alja Zottel, Neja Šamec, Marija Skoblar Vidmar, Alenka Matjašič, Andrej Zupan, Jernej Mlakar, Maria Suntsova, Denis V. Kuzmin, Anton Buzdin, Ivana Jovčevska

**Affiliations:** 1 European Organization for Research and Treatment of Cancer (EORTC), Biostatistics and Bioinformatics Subgroup, 1000 Brussels, Belgium; sorokin_m_i@staff.sechenov.ru; 2 World-Class Research Center “Digital Biodesign and Personalized Healthcare”, Sechenov First Moscow State Medical University, 119991 Moscow, Russia; suntsova_m_v@staff.sechenov.ru; 3 Moscow Institute of Physics and Technology, National Research University, 141700 Moscow, Russia; raevskii.mm@phystech.edu (M.R.); denisk@list.ru (D.V.K.); 4 Medical Centre for Molecular Biology, Institute of Biochemistry and Molecular Genetics, Faculty of Medicine, University of Ljubljana, 1000 Ljubljana, Slovenia; alja.zottel@mf.uni-lj.si (A.Z.); neja.samec@mf.uni-lj.si (N.Š.); 5 Institute of Oncology, 1000 Ljubljana, Slovenia; mskoblar@onko-i.si; 6 Institute of Pathology, Faculty of Medicine, University of Ljubljana, 1000 Ljubljana, Slovenia; alenka.matjasic@mf.uni-lj.si (A.M.); andrej.zupan@mf.uni-lj.si (A.Z.); jernej.mlakar@mf.uni-lj.si (J.M.); 7 Shemyakin-Ovchinnikov Institute of Bioorganic Chemistry, Russian Academy of Sciences, 117997 Moscow, Russia; 8 OmicsWay Corp., Walnut, CA 91789, USA

**Keywords:** glioblastoma, noncoding RNA, *CRNDE*, RNA sequencing, long-term survival

## Abstract

**Simple Summary:**

Most glioblastoma patients succumb to the disease within 12 to 18 months, and only 9% are alive 2 years after diagnosis. Even with extensive research, the life expectancy of glioblastoma patients has not changed in decades. We aimed to identify differences in the transcriptomic profiles of glioblastoma patients with long and short survival. With large-scale transcriptomic analysis, we examined information from publicly available datasets (TCGA and CGGA) in combination with FFPE patient tissue samples. We identified one gene, the long noncoding RNA *CRNDE*, whose overexpression is directly correlated with poor patient survival. Therefore, we suggest its further confirmation as a negative prognostic glioblastoma biomarker. Glioblastoma management still lacks suitable diagnostic, predictive, and prognostic biomarkers for early disease diagnosis, and treatment follow-up. We believe our findings can serve as the basis for identification of new and potential suitable disease biomarkers by looking beyond the classical molecules (DNA, RNA, and proteins) into the noncoding genome.

**Abstract:**

Glioblastoma is the most common and malignant brain malignancy worldwide, with a 10-year survival of only 0.7%. Aggressive multimodal treatment is not enough to increase life expectancy and provide good quality of life for glioblastoma patients. In addition, despite decades of research, there are no established biomarkers for early disease diagnosis and monitoring of patient response to treatment. High throughput sequencing technologies allow for the identification of unique molecules from large clinically annotated datasets. Thus, the aim of our study was to identify significant molecular changes between short- and long-term glioblastoma survivors by transcriptome RNA sequencing profiling, followed by differential pathway-activation-level analysis. We used data from the publicly available repositories The Cancer Genome Atlas (TCGA; number of annotated cases = 135) and Chinese Glioma Genome Atlas (CGGA; number of annotated cases = 218), and experimental clinically annotated glioblastoma tissue samples from the Institute of Pathology, Faculty of Medicine in Ljubljana corresponding to 2–58 months overall survival (n = 16). We found one differential gene for long noncoding RNA *CRNDE* whose overexpression showed correlation to poor patient OS. Moreover, we identified overlapping sets of congruently regulated differential genes involved in cell growth, division, and migration, structure and dynamics of extracellular matrix, DNA methylation, and regulation through noncoding RNAs. Gene ontology analysis can provide additional information about the function of protein- and nonprotein-coding genes of interest and the processes in which they are involved. In the future, this can shape the design of more targeted therapeutic approaches.

## 1. Introduction

Glioma incidence has been steadily rising over recent decades, and the mortality curve follows the same trend [1]. World Health Organization (WHO) grade IV astrocytoma otherwise known as glioblastoma is the most common and accounts for 60–70% of all glioma cases [2]. Because of unspecific symptoms such as headache, confusion, and hearing or vision problems, glioblastoma is usually diagnosed in advanced stage.

Clinical presentation is typically short and ranges from 3 to 6 months before diagnosis [3]. The most widely accepted standard of care is the Stupp protocol that consists of maximal surgical resection, 6 weeks radiation and daily oral temozolomide chemotherapy followed by six subsequent cycles of adjuvant temozolomide [4]. Such aggressive treatment prolongs patient survival up to 12–18 months after diagnosis [5]. As low as 9% of patients are alive 2 years after diagnosis [6], while only 0.7% of all glioblastoma patients survive more than 10 years after diagnosis [7].

Glioblastomas are divided into two main groups primary or isocitrate dehydrogenase (*IDH*) wild-type and secondary or *IDH* mutant [8,9]. The Cancer Genome Atlas (TCGA) project provided the first comprehensive analysis of the molecular profile of glioblastoma [10]. Later, by molecular profiling, three main glioblastoma subtypes were defined [11]. Moreover, glioblastoma presents with a whole spectrum of genetic and epigenetic changes as well as whole or partial chromosome gains or losses, and transcriptional interference [12].

Currently there are no available biomarkers for diagnosing glioblastoma in early disease stages or for monitoring patient performance [13]. In recent years, several different molecules have been proposed as putative imaging or molecular biomarkers [14,15,16,17,18,19]. So far, only two predictive biomarkers have been identified, namely O^6^-methylguanine DNA methyltransferase (*MGMT*) promoter methylation [20,21] and chromosome 1p/19q codeletion [22,23], that predict positive response to therapy and longer survival in elderly patients. Dismal patient prognosis means new methods need to be applied for identification of promising molecular biomarkers for diagnosis and prediction of treatment response.

In this study, we performed large-scale transcriptomic profiling to identify differences between glioblastoma patients with relatively short- and long-term overall and progression-free survival (OS and PFS, respectively). We found the long noncoding RNA *CRNDE* as differentially expressed i.e., overexpressed in all investigated glioblastoma datasets. Because the overexpression of *CRNDE* was significantly associated with poor OS and PFS, we conclude it shows the potential to be used as a negative prognostic biomarker for glioblastoma. Still, its possible implication in clinical practice as a prognostic biomarker needs to be further experimentally elaborated.

## 2. Materials and Methods

### 2.1. Ethics Statement

This study was carried out in accordance with The Code of Ethics of the World Medical Association (Declaration of Helsinki) for experiments involving human samples. The manuscript is in line with the Recommendations for the Conduct, Reporting, Editing and Publication of Scholarly Work in Medical Journals and aim for the inclusion of representative human populations (sex, age and ethnicity) as per those recommendations.

The use of biological tissue samples in the study was approved by the National Medical Ethics Committee of the Republic of Slovenia approval numbers 0120-196/2017/7, 0120-190/2018/4 and 0120-190/2018/11. All patients signed written informed consent. Samples used in this study are anonymous.

### 2.2. Biosamples

In this study, we used 16 formalin-fixed, paraffin-embedded (FFPE) primary tumor tissue samples from glioblastoma patients. FFPE samples were obtained from the Institute of Pathology, Faculty of medicine, University of Ljubljana. FFPE samples contained from 50% (1 sample), 70% (3 samples), 80% (5 samples) and 90% (7 samples) cancer cells in the specimens. Patients were treated at the Institute of Oncology, Ljubljana. Samples were collected retrospectively from patients admitted between the years 2012 and 2017. The patient’s age at diagnosis ranged from 22 to 70 years old, 11 (68.75%) of patients were man, 5 (31.25%) were women. The patient’s PFS defined as the time from first diagnosis to glioblastoma recurrence varied in the range of 2–37 months, OS varied between 2 and 58 months. More detailed clinicopathological information is shown in Table 1. 

### 2.3. RNA Isolation and Sequencing

RNA libraries were generated, sequenced, and processed as previously described [24]. RNA extraction was performed using RecoverAll™ Total Nucleic Acid Isolation Kit (Invitrogen, New York, NY, USA), and RNA Integrity Number (RIN) was measured with Agilent 2100 bioanalyzer (Agilent Technologies, Inc., Santa Clara, CA, USA). RNA concentrations were measured using Qubit RNA Assay Kit (Life Technologies Limited, Paisley, UK), and ribosomal RNA was depleted using RNA Hyper Kit with RiboErase (KAPA Biosystem, Roche, Cape Town, South Africa). Processed library concentrations and length distributions were measured using Qubit ds DNA HS Assay kit (Life Technologies Limited, Paisley, UK) and Agilent Tapestation (Agilent Technologies, Inc., Santa Clara, CA, USA), respectively. The samples were sequenced using Illumina HiSeq 3000 engine (Illumina Inc., Hayward, CA, USA) for single end sequencing, 50 bp read length, for approximately 30 million raw reads per sample. Data quality check was conducted using Illumina SAV. De-multiplexing was performed using Illumina Bcl2fastq2 v 2.17 software.

### 2.4. RNA Sequencing Data Collection and Processing

Non-experimental RNA sequencing profiles of primary glioblastoma specimens annotated with known overall survival (OS) and progression-free survival (PFS) time were collected from TCGA [25] and CGGA [26] databases. In total we took 135 samples from TCGA and 218 samples from CGGA database. Data from TCGA, CGGA, and current experimental dataset were used in raw gene counts format. Totally, expression levels were established for 36,596 annotated human genes in TCGA, 23,271 genes in CGGA and 37,002 genes in the experimental dataset. Raw gene counts were normalized using quantile normalization method [27] and log_10_-transformed for further analyses.

### 2.5. Quantization of Molecular Pathway Activation

Pathway activation levels (PALs) were established using Oncobox pathway analysis method [28] for 1611 molecular pathways containing 10 or more gene products extracted from the public databases [29] using the original software [29]. For PAL calculations, each sample expression profile was normalized on mean geometrical levels of gene expression for all samples in the dataset under investigation.

### 2.6. Survival Analysis

Each gene and molecular pathway were assessed separately to interrogate their possible impact on PFS and OS. For the analysis of each database, we included patients of the following two groups based on their gene expression or PAL: (*i*) group with gene expression or PAL higher than 66th-percentile, (*ii*) group with gene expression or PAL lower than 33rd-percentile.

Cox survival analysis for OS and PFS was performed between those two groups using R packages survival [30] and survminer [31]. Then log-rank *p*-value and hazard ratio *p*-value were calculated. Genes and molecular pathways with both *p*-values below 0.05 threshold were selected for further analyses.

### 2.7. Plots and Visualizations

Principal component analysis (PCA) and visualization were done for log-transformed quantile normalized gene counts using *prcomp* and *pca2d* R (v.3.6.0) functions. Venn diagrams were drawn using R package *venn* (v.1.10) [32]. Kaplan–Meier plots were drawn using *ggsurvplot* (*survminer* package v.0.4.9) R function [31], tables with hazard ratio, confidence intervals and *p*-values were drawn using *ggforest* (*survminer* package v.0.4.9) R function [32,33]. Volcano plots for hazard ratio were visualized using R package EnhancedVolcano (v. 1.10.0) [34].

### 2.8. Testing of Intersections Significance

To test whether a given number of common differential genes or pathways between the two of three intersecting datasets is significant, 1000 random intersections were performed according to [33]. In every case, two/three random samples from two/three corresponding gene sets of the respective datasets were taken. Then these random samples were intersected for each iteration and 1000 numbers of random common genes were obtained. *p*-value of intersection significance was calculated as a fraction of random numbers equal or higher than the experimentally observed number of common genes.

### 2.9. Data and Code Availability

Sequencing data were deposited in NCBI Sequencing Read Archive (SRA) under accession ID PRJNA742887. Code for the data analysis can be found on Gitlab [35].

## 3. Results

### 3.1. RNA Sequencing of Experimental Glioblastoma Samples

In this study, samples from 16 glioblastoma patients (Table 1) were enrolled to obtain experimental RNA sequencing profiles annotated with survival data. In this group there were 11 males, 5 females, age range was 22–70 y.o., mean age was 55.75 y.o. The minimal Karnofsky Performance Status (KPS) at the time of diagnosis in this group was 50, median KPS was 90. Overall survival (OS) data were available for 15 patients (range 2–58 months, mean OS 21.1 months) after diagnosis i.e., the date of the surgical resection. Progression-free survival (PFS) data were available for 14 patients (range 2–37 months, mean PFS 13.2 months). The tumor samples were formalin-fixed, paraffin-embedded tumor tissue blocks with at least 50% of tumor cells. We then profiled gene expression in these biosamples by RNA sequencing (RNAseq) and obtained 26.36–38.99 million raw reads per library (mean 32.68 million reads). A range of 5.53–11.09 million reads (mean 7.29) was uniquely mapped to Ensembl genes using STAR aligner (Table 2). Two samples, NB-00339/13 and NB-00046/12, were not of enough quality.

### 3.2. Primary Comparison of RNAseq Profiles among the Experimental and Literature Datasets

To overall characterize the data obtained, we compared by principal component analysis (PCA) distributions of RNAseq profiles among the experimental dataset (*n* = 16) and publicly available datasets from The Cancer Genome Atlas (TCGA) project database; number of annotated cases = 135, and from Chinese Glioma Genome Atlas (CGGA) project database; *n* = 218. TCGA profiles were annotated with OS and PFS data, and CGGA profiles with only OS data.

**Figure 1:**
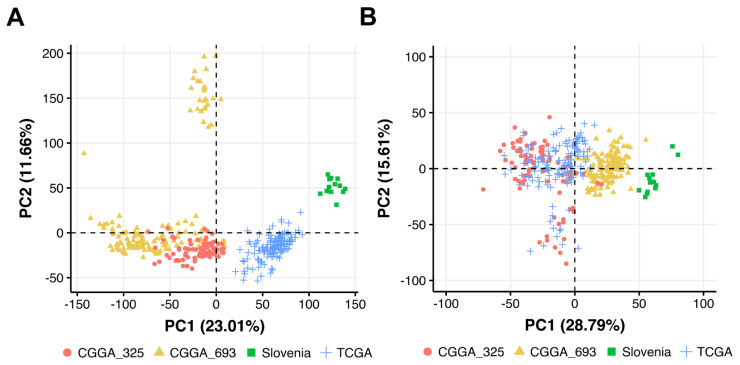
Principal Component Analysis (PCA) of (**A**) gene expression and (**B**) pathway activation levels (PALs) for experimental (Slovenia) clinically annotated glioblastoma tissue samples and publicly available glioblastoma samples from CGGA (batches CGGA_325 and CGGA_693) and TCGA.

PCA was performed in the space of log_10_ transformed quantile normalized gene counts. We found that CGGA-derived samples formed two overall distinct clusters that corresponded to two different batches with different biomaterial treatment and sequencing protocols (Figure 1A). TCGA and experimental (termed as “Slovenia”) samples also formed distinct clusters according to each experimental platform (Figure 1A).

We then built PCA plot based on 1611 molecular pathway activation profiles [29], where *pathway activation level* (PAL) of a pathway is calculated using transcriptomic data (Figure 1B). PAL can take positive or negative values in the case of pathway up- or down-regulation, respectively, and positively reflects the extent of a pathway activation, and thus can be used as the quantitative characteristic of the interactome under study [36].

On the 1611-pathway PCA plot, we observed the same trend as on the gene-based figure, thus demonstrating that CGGA-derived samples from different batches (CGGA_325 and CGGA_693) should be regarded as two separate datasets (Figure 1B).

### 3.3. Survival-Linked Differential Gene Analysis

For each dataset under investigation we performed Cox survival analysis and extracted relevant differential genes [37]. To this end, for every gene we identified two respective patient groups by this gene expression levels: (*i*) patients with the expression level higher than 66th-percentile among all patients (in the top third), and (*ii*) patients with the expression level less than 33rd-percentile (in the bottom third). We then performed Cox-regression analysis for OS and PFS data between these groups according to the previous protocol [38,39].

**Figure 2:**
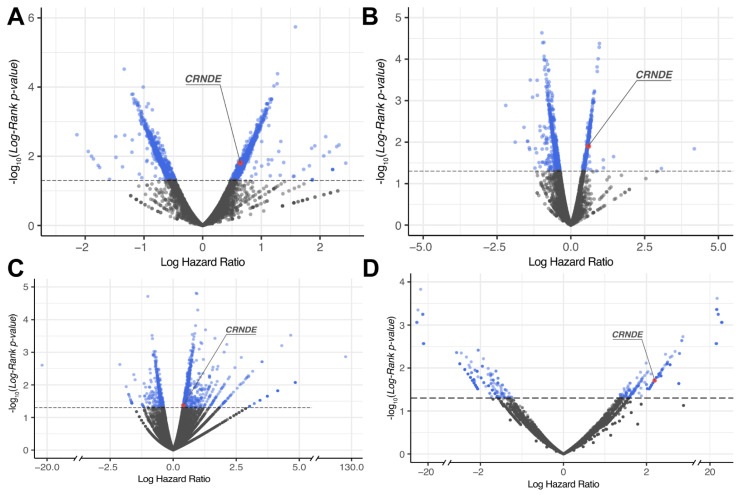
Distribution of differential genes by log-rank *p*-value and log hazard ratio for overall survival (OS) data across: (**A**) CGGA_325, (**B**) CGGA_693, (**C**) TCGA and (**D**) Slovenia glioblastoma datasets.

**Figure 3:**
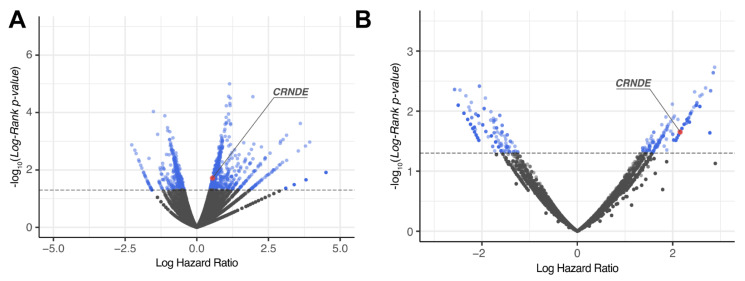
Distribution of differential genes by log-rank *p*-value and log hazard ratio for progression-free survival (PFS) data across: (**A**) TCGA and (**B**) Slovenia glioblastoma datasets.

For each gene, log-rank *p*-value and hazard ratio (HR) *p*-value were obtained and then genes with both *p*-values less than 0.05 were considered differential [40] (Figure 2 and Figure 3 and Supplementary Table S1). The differential genes obtained were then intersected between the TCGA, CGGA (separately CGGA_325 and CGGA_693 batches), and the experimental (Slovenia) datasets.

**Figure 4:**
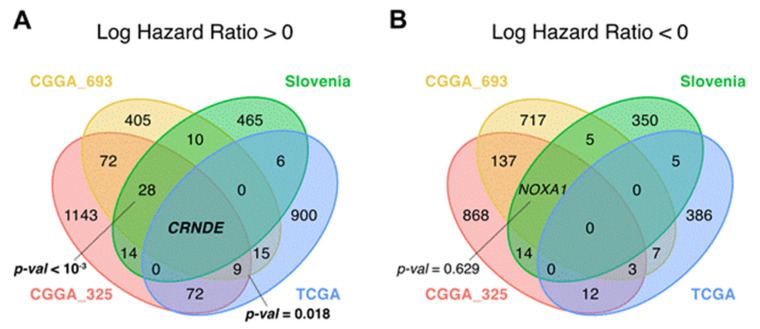
Intersections of differential (**A**) *plus* (hazard ratio > 0) and (**B**) *minus* (hazard ratio < 0) genes for overall survival (OS) analysis between CGGA_325, CGGA_693, TCGA and the experimental (Slovenia) glioblastoma samples; *p*-values for intersection significance that are less than 0.05 are highlighted in bold.

**Figure 5:**
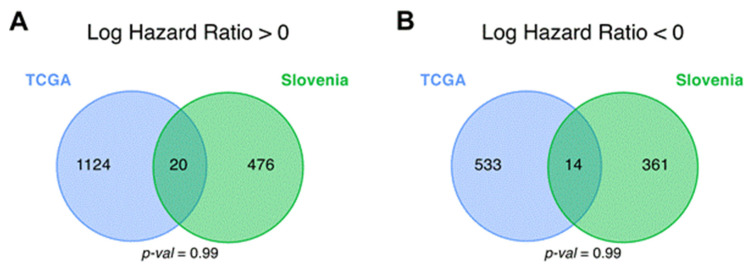
Intersections of differential (**A**) *plus* (hazard ratio > 0) and (**B**) *minus* (hazard ratio < 0) genes for progression-free survival (PFS) analysis between CGGA_325, CGGA_693 and TCGA glioblastoma samples.

The genes that were statistically significantly positively (HR > 0), and negatively (HR < 0) associated with OS (termed *plus* and *minus* genes, respectively) were considered separately. Totally, we found 1003 *plus*/413 *minus* genes for the TCGA, 1331/1670 genes for the CGGA, and 502/377 genes for the experimental datasets, respectively. We then intersected the gene sets obtained and found only one common gene that was differentially regulated (overexpressed) in all four datasets (Figure 4 and Figure 5, Supplementary Table S2). This was the gene for noncoding RNA *CRNDE* (ColoRectal Neoplasia Differentially Expressed) that was previously associated with many cancers [41,42] including glioblastoma [43,44]. 

To test if a given number of common differential genes or pathways between intersecting datasets is statistically significant, perturbation test for randomness was performed according to [33] (see Materials and Methods). Briefly, we selected random sets of genes/pathways from all available genes/pathways and repeated this operation 1000 times. The percentile of the observed intersection in the distribution of random intersections was considered to be a measure of statistical significance. For OS data, we found that the intersections of the *plus* genes in TCGA vs. CGGA comparison, and in CGGA vs. Slovenia dataset comparison were non-random, because they returned statistically significantly greater number of intersecting genes as for the random intersection model (Figure 4 and Supplementary Table S2). 

**Figure 6:**
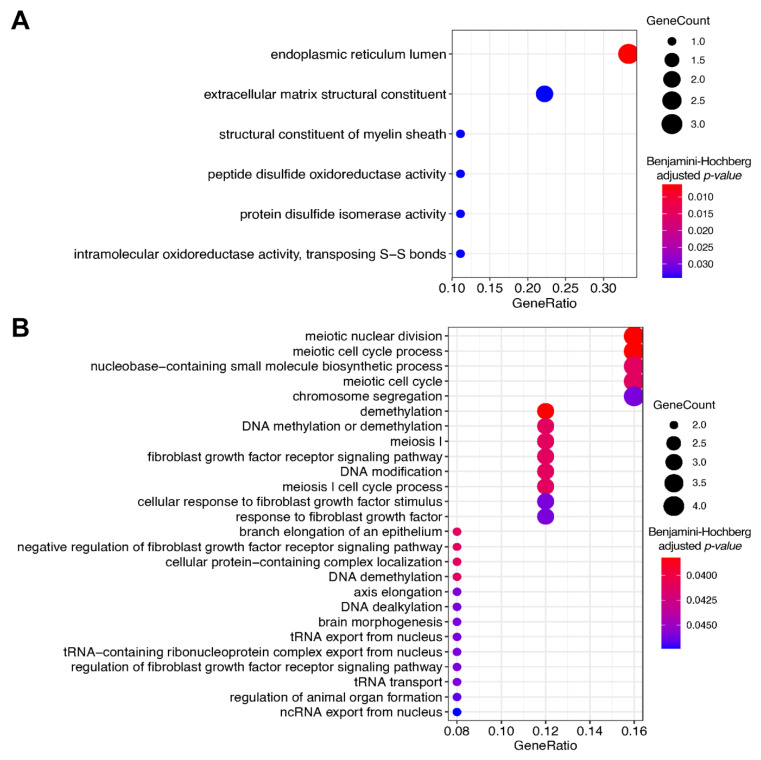
Gene Ontology analysis of differential *plus* genes (linked with hazard ratio > 0 in overall survival analysis) that were statistically significantly intersected between (**A**) CGGA_325, CGGA_693, and TCGA; (**B**) CGGA_325, CGGA_693, and Slovenia datasets.

We then investigated these non-random intersecting gene sets using Gene Ontology (GO) terms analysis [45] and identified enriched biological processes associated with these gene sets (Figure 6). For statistical estimates, we used Benjamini–Hochberg method for false discovery rate (FDR) correction [46] and *p*-value threshold 0.05 [40].

For *plus* genes that were in statistically significant intersection of TCGA, CGGA_325, and CGGA_693 datasets, the most strongly enriched GO terms were linked with endoplasmic reticulum lumen and structure of extracellular matrix (Figure 6A and Supplementary Table S3). In turn, for the statistically significant intersection of CGGA_325, CGGA_693 and Slovenia *plus* genes, the most strongly enriched terms dealt with the processes of meiosis and chromosomal segregation, DNA demethylation, fibroblast growth factor receptor signaling, and tRNA transport from the nucleus (Figure 6B and Supplementary Table S4).

We then assessed associations of gene expression biomarkers with progression-free survival (PFS) data. PFS was annotated only for the TCGA and Slovenia datasets, but no information could be extracted for both CGGA batches. Similar to OS expression biomarker analysis, we screened for differentially regulated genes (Figure 3 and Figure 5) using the same analytic approaches and settings. However, for both *plus* and *minus* genes, the intersections observed did not pass the randomness permutation test, as reflected by high *p*-values (Figure 5).

### 3.4. Differential Pathway Activation Analysis

**Figure 7:**
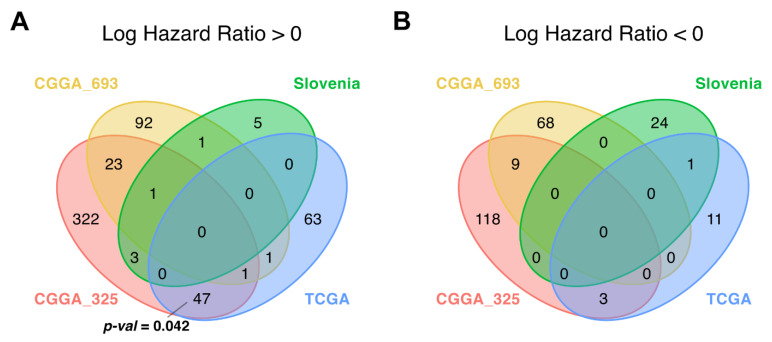
Intersections of differential (**A**) *plus* (hazard ratio > 0) and (**B**) *minus* (hazard ratio < 0) molecular pathways for overall survival (OS) analysis between CGGA_325, CGGA_693, TCGA and the experimental (Slovenia) glioblastoma expression datasets; *p*-values for intersection significance that are less than 0.05 are highlighted in bold.

When performing differential pathway-activation-level (PAL) analysis for the same three datasets and overlapping the results with the same settings as for the individual genes, we identified non-random intersection pattern only for the *plus* pathways between CGGA and TCGA datasets (*p* = 0.042; Figure 7A, Supplementary Tables S2, S4 and S5).

We totally identified 47 (CGGA, TCGA) common differential *plus* pathways (Supplementary Table S2). In good agreement with the results of individual gene-level analysis (Figure 4), they were mostly dealing with the regulation of extracellular matrix maintenance (Supplementary Table S5). However, there were no triple intersections, and we could not identify consensus biomarker molecular pathway(s) for OS in glioblastoma patients. There were also no *minus* pathways that would pass the randomness permutation test (Figure 7B and Supplementary Table S2).

**Figure 8:**
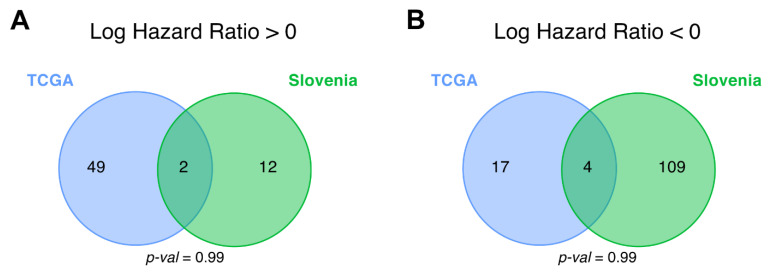
Intersections of differential (**A**) *plus* (hazard ratio > 0) and (**B**) *minus* (hazard ratio < 0) molecular pathways for progression-free survival (PFS) analysis between CGGA_325, CGGA_693, TCGA and the experimental (Slovenia) glioblastoma samples.

When screening for the PFS biomarkers, we also could not identify statistically significant interactions, for both *plus* and *minus* pathways (Figure 8).

### 3.5. CRNDE Overexpression Is Associated with Poor Patient Overall Survival

**Figure 9:**
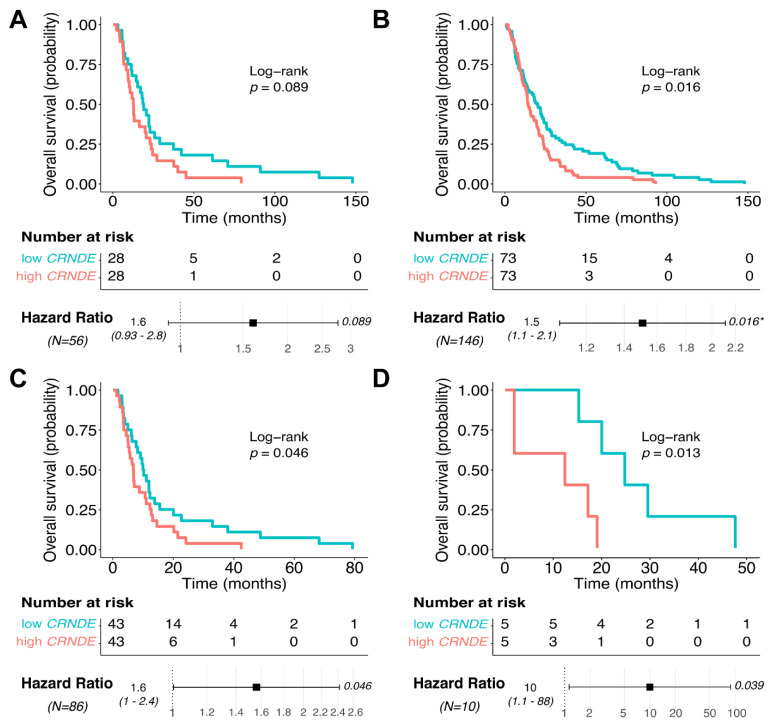
Kaplan–Meier plot and hazard ratio for *CRNDE* expression in glioblastoma samples across (**A**) CGGA_325, (**B**) CGGA_693, (**C**) TCGA and (**D**) Slovenia datasets for overall survival (OS) analysis. These results demonstrate that in there out of four tested datasets with available overall survival data (CGGA_693, TCGA, Slovenia), glioblastoma patients with overexpressed *CRNDE* had significantly lower overall survival (*p* = 0.013–0.046).

Thus, we identified that *CRNDE* was the unique consensus OS expression biomarker among the glioblastoma datasets tested; however, for PFS no common expression biomarkers were identified. We then separately analyzed the impact of *CRNDE* expression on glioblastoma patient OS using the clinical information from the same annotated expression datasets (Figure 9). High *CRNDE* expression was significantly associated with poor OS in CGGA_693, TCGA, and Slovenia datasets (Figure 9B–D). The same trend was observed for CGGA_325 dataset; however, the survival difference did not reach *p*-value threshold of 0.05 (Figure 9A).

**Figure 10:**
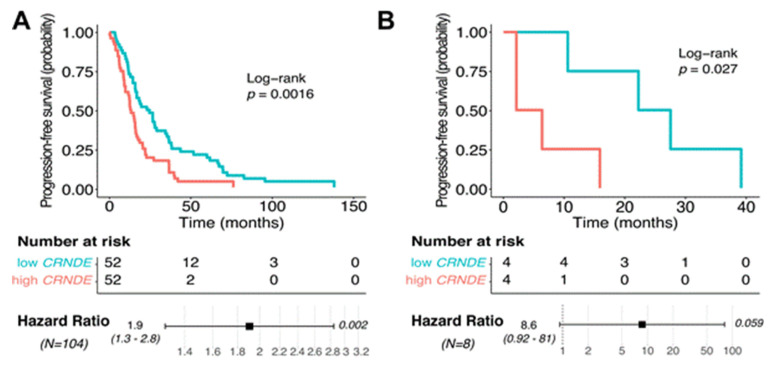
Kaplan–Meier plot and hazard ratio for *CRNDE* expression in glioblastoma samples across (**A**) TCGA and (**B**) Slovenia datasets for progression–free survival (PFS) analysis.

We observed a similar trend of *CRNDE* expression being positively associated with poor survival also for the PFS (Figure 3 and Figure 10). Log-rank *p*-values were good enough for both TCGA (*p* = 0.0016) and experimental (Slovenia; *p* = 0.027) datasets (Figure 10). Hazard ratio *p*-value was good for the TCGA (*p* = 0.002) but borderline (*p* = 0.059) for the experimental dataset (Figure 10).

Thus, we conclude that increased *CRNDE* expression can be indicative of poor OS and PFS in glioblastoma.

## 4. Discussion

In years of life lost, primary glioblastomas are ranked first among cancer types—on average 20.1 years compared to 11.8 years for lung cancer and 6.8 years for prostate cancer [12]. This is partially because of the lack of molecular biomarkers for early disease diagnosis and treatment follow-up. Because of the high mortality rate, discovery of molecular biomarkers for glioblastoma is one of the priorities in neuro-oncology research. With our study we aimed to exploit the potential of high throughput in silico tools to identify putative biomarkers to clinically manage glioblastoma.

To obtain more information about the molecular differences between glioblastoma patients with relatively long- and short-term survival, we performed transcriptomic analysis. We combined data already available from public repositories of TCGA and CGGA projects with the original data obtained for clinically annotated experimental FFPE tissue samples from glioblastoma patients. As shown on Table 1, the patients that were included in this study had represented both short- and long-term survival glioblastoma groups. We could identify one gene, *CRNDE*, which expression has the potential to be used as a negative prognostic biomarker of patient survival, for both OS and PFS. Specifically, Kaplan–Meier analysis revealed that patients who had increased *CRNDE* expression levels generally had shorter survival times. Moreover, Gene Ontology and pathway-activation-level analysis revealed cell migration, reshaping of extracellular matrix, meiosis, and chromosomal segregation, FGFR signaling, tRNA/noncoding RNA transfer from nucleus, and DNA demethylation as the major differential processes between the long and poor survivors in glioblastoma.

### CRNDE

*Colorectal neoplasia differentially expressed* (*CRNDE*) was discovered first as overexpressed gene in colorectal adenomas and adenocarcinomas [47]. Since that it was associated with different human malignancies [48] including glioblastoma [49,50]. *CRNDE* is located on chromosome 16 [41] and is transcribed to form multiple RNA transcripts, some of which function as noncoding regulatory RNAs. Among them *CRNDE*-a, -b, -d, -e, -f, -h and -j are thought to be major transcripts in different cancers [42]. These noncoding RNAs can regulate gene expression [51,52].

Most of the transcribed human genome is noncoding and contains small (sRNAs) and long noncoding RNAs (lncRNAs) [53]. lncRNAs vary in length from 200 bp to over 100 kb [54]. In humans, almost 40% of lncRNAs are specific to the brain [55,56] that most probably reflects its regulatory complexity [57]. Many brain-specific lncRNAs are evolutionary conserved, and are believed to have important functions in brain development and functioning [58]. A correlation between glioblastoma and various lncRNAs has been established in attempts for better glioma sub-classification [58,59,60,61,62] with relation to tumor initiation [61,63], progression [56,58,61,62] or treatment resistance [64,65,66,67,68] on multiple occasions.

Because of their tissue specificity lncRNAs are excellent candidates for the design and development of targeted therapeutics. However, in order to achieve this one has to identify disease-relevant signaling pathways and their druggable targets, and then to find a way to genetically manipulate them as well as to find an effective delivery system [69].

Other studies have also established a correlation between *CRNDE* and glioblastoma. For example, by analyzing a cohort of previously published glioma gene expression profiles from the Gene Expression Omnibus (GEO) Zhang et al. [70] showed that expression of lncRNAs *CRNDE* and *HOTAIR* increases with ascending glioma malignancy grade. A small scale preliminary study by Kiang et al. [71] showed that different transcript variants of *CRNDE* have clinical impact in glioblastoma. Although the authors did not show a direct link between a specific *CRNDE* transcript variant and patient survival, they observed a trend between the ratio of two variants and patient survival.

By analyzing 19 astrocytomas, of which 5 were low grade and 14 were high grade tumors, Kiang et al. [72] showed that *CRNDE* is strongly up-regulated in gliomas and positively correlates with epidermal growth factor receptor (EGFR) amplification. Moreover, the authors showed that *CRNDE* knockdown suppresses glioma cell growth in vitro and in vivo, and is associated with decreased Bcl2/Bax ratio. Its potential to be used as a predictive marker was published by Jing et al. [73] in a retrospective study. The authors performed qRT-PCR to examine *CRNDE* expression and established association between high *CRNDE* expression and clinicopathological features such as larger tumor size, higher WHO grade, and recurrence.

In gliomas, *CRNDE* can affect proliferation, apoptosis, inflammation, invasion and migration [41]. In particular, its overexpression promotes proliferation, migration and invasion, and inhibits apoptosis [43,74]. Still, the functional roles of lncRNAs, and especially *CRNDE* for glioblastoma, must be experimentally validated. There are some indications that *CRNDE* can promote glioma malignancy through activation of EGFR signaling [72] and miR-384/PIWIL4/STAT3 axis [74], by preventing miR-136-5p-mediated down-regulation of Bcl-2 and Wnt2 [75] and mTOR [76] pathways, and can also trigger inflammation through the TLR3-NF-κB-cytokine signaling pathway [77]. In addition, Zheng et al. [43] showed that *CRNDE* positively regulates *X-linked inhibitor of apoptosis* (*XIAP*) and *serine/threonine protein kinase PAK 7* (*PAK7*) through miR-186 in glioblastoma stem cells. Although the mechanism of action remains to be determined, lncRNAs are a source for identification of novel biomarkers for glioblastoma diagnosis or therapy-guidance.

It is clear now that lncRNAs play critical roles in many pathologies and they are the forefront molecules for translational research. Because of its obvious overexpression in tumors, *CRNDE* is one of the most extensively investigated lncRNAs in cancer research. The major novelty of our study is that we performed RNA sequencing profiling instead of the most commonly used microarray gene expression analysis. Additionally, we used a combination of data from previous repositories and from the experimental cohort consisting of short- and long-term glioblastoma survivors. Our methodology proved successful in the identification of the lncRNA *CRNDE* as a negative prognostic biomarker. Although other studies linked *CRNDE* with increased malignancy grade, we confirmed here for the first time that *CRNDE* overexpression directly correlates with shorter patient survival times. Thus, we propose its further detailed investigation as a prognostic biomarker for life expectancy of glioblastoma patients.

## 5. Limitations

The relatively small number (*n* = 16) of experimental clinically annotated samples we used in our study can be considered to be a limitation. Our initial aim was to collect samples from long-term glioblastoma survivors. Because of the low incidence of glioblastoma and poor patient survival it is difficult to obtain large number of such samples in countries with small population. Still, to provide more scientifically solid results, the experimental data were complemented by publicly available transcriptomic data. The results may vary between the databases due to different patient ethnicities and/or countries’ clinical guidelines for diagnosis and therapy.

Another limitation is that we did not include other potential prognostic factors such as extent of the surgical resection in our analysis. Therefore, we reason it is crucial to additionally investigate the role of *CRNDE* in respect to other prognostic factors in glioblastoma before determining its potential clinical application.

## 6. Conclusions

Despite decades of extensive research and implementation of unconventional therapeutic approaches, long-term survival is not common for glioblastoma patients. Identification of diagnostic as well as prognostic and predictive therapeutic biomarkers is therefore crucial. This can be initially achieved by large-scale profiling combined with extensive bioinformatics analysis, and further confirmed experimentally before it reaches clinical settings. As shown in our study, transcriptome profiling by RNA sequencing is an appropriate method for identification of biomarkers with potential clinical utility. We analyzed pathway activation levels of intersecting genes between TCGA, CCGA, and experimental dataset to determine their possible impact on OS and PFS. Using Cox survival analysis and intersection of positive and negative differential genes between datasets, we identified only one common gene, *CRNDE*, that was negatively correlated with OS. With Kaplan–Meier analysis we confirmed that overexpression of *CRNDE* can be used as an indication of poor OS and PFS. As suggested, lncRNA *CRNDE* can be used as a negative prognostic marker for glioblastoma patients.

## Figures and Tables

**Table 1:** Clinicopathological information about experimental glioblastoma patients involved in this study.

Sample ID	Gender	Age at Diagnosis	KPS	WHO Grade	Recurrent Tumor	OS (Months)	*IDH1* R132H Mutation	*ATRX* Loss	*p53*	*MGMT* Methylation	GTR	Therapy	Adjuvant Therapy	TTP (Months)
NB-00131/12	M	42	90	IV	No	13	Negative	Not performed	Negative	No	No	60 Gy radiotherapy + TMZ	Adjuvant TMZ	/
NB-00094/13	M	47	90	IV	No	16	Negative	Not performed	Positive	Yes	Yes	60 Gy radiotherapy + TMZ	Adjuvant TMZ	10
17-H-21518	M	64	100	IV	No	16	Negative	No	Negative	No	Yes	60 Gy radiotherapy + TMZ	Adjuvant TMZ	8
NB-00233/12	F	42	100	IV	No	18	Negative	Not performed	Negative	No	Yes	60 Gy radiotherapy + TMZ	No (TMZ side effects)	6
NB-00464/12	M	62	90	IV	No	19	Not performed	Not performed	Not performed	No	No	30 Gy radiotherapy + TMZ	Adjuvant TMZ	14
NB-00079/14	M	43	90	IV	No	20	Positive	Not performed	Positive	No	Yes	60 Gy radiotherapy + TMZ	Adjuvant TMZ	15
17-H-05191	M	68	100	IV	No	21	Negative	No	Positive	No	Yes	60 Gy radiotherapy + TMZ	Adjuvant TMZ	12
16-H-17976	M	66	70	IV	No	23	Negative	No	Negative	Yes	Yes	60 Gy radiotherapy + TMZ	Adjuvant TMZ	16
NB-00173/14	M	55	90	IV	No	26	Negative	Not performed	Negative	Yes	Yes	60 Gy radiotherapy + TMZ	Adjuvant TMZ	21
NB-00369/13	F	62	90	IV	No	31	Negative	Not performed	Positive	No	Yes	60 Gy radiotherapy + TMZ	Adjuvant TMZ	26
NB-00450/12	F	58	80	IV	No	50	Negative	Not performed	Negative	Yes	No	60 Gy radiotherapy + TMZ	Adjuvant TMZ	37
NB-00038/13	F	61	80	IV	No	58	Negative	Not performed	Positive	No	Yes	60 Gy radiotherapy + TMZ	Adjuvant TMZ	14
NB-00339/13	M	22	100	IV	No	91	Negative	Not performed	Negative	No	Yes	60 Gy radiotherapy + TMZ	Adjuvant TMZ	90 *
NB-00046/12	M	62	80	IV	No	2	Negative	Not performed	Negative	Yes	No	34 Gy radiotherapy + TMZ	No	2
NB-00003/15	F	68	70	IV	No	2	Negative	Yes	Negative	No	No	30 Gy radiotherapy	/	2
17-H-31914	M	70	50	IV	No	2	Negative	No	Negative	Yes	No	None	None	2

KPS: Karnofsky performance scale; WHO: World Health Organization; *IDH1*: Isocitrate dehydrogenase 1; R: Arginine; H: Histidine; *ATRX*: ATP-dependent helicase ATRX, X-linked helicase II; *p53*: Tumor protein p53; *MGMT*: O^6^-methylguanine DNA methyltransferase promoter methylation status; GTR: Gross total resection; TTP: Time to progression; OS: overall survival; TMZ: Temozolomide; *: Patient presented with progression after the analysis was performed.

**Table 2:** RNA sequencing statistics.

Sample ID	Library ID	Uniquely Mapped Reads	Total Reads
NB-00369/13	GB_1	6.57	38.16
17-H-21518	GB_2	6.56	32.52
NB-00464/12	GB_3	11.09	36.74
NB-00079/14	GB_4	5.85	28.14
17-H-05191	GB_5	7.18	38.79
16-H-17976	GB_6	6.19	34.93
NB-00173/14	GB_7	5.80	38.31
NB-00131/12	GB_8	10.55	38.99
NB-00450/12	GB_10	8.36	28.62
NB-00450/12	GB_11	5.84	26.36
NB-00233/12	GB_12	6.97	30.42
NB-00003/15	GB_16	5.53	28.93
17-H-31914	GB_17	7.10	28.03
NB-00094/13	GB_18	8.52	28.62

## Data Availability

Sequencing data were deposited in NCBI Sequencing Read Archive (SRA) under accession ID PRJNA742887. Code for the data analysis can be found on Gitlab at the following link: https://github.com/raevskymichail/CRNDE_glioblastoma/tree/v0.1 (accessed on 25 June 2021).

## Supplementary Materials

The following are available online at https://www.mdpi.com/article/10.3390/cancers13143419/s1, Table S1: HR and log-rank *p*-value for gene expression levels in CGGA, TCGA, and Slovenia datasets, Table S2: Intersections of differentially expressed genes and pathways, Table S3: Common *plus* genes that were in statistically significant intersection of TCGA, CGGA_325, and CGGA_693 datasets, Table S4: Common *plus* genes that were in statistically significant intersection of Slovenia, CGGA_325, and CGGA_693 datasets, Table S5: common *plus* pathways that were in statistically significant intersection of CGGA_325 and TCGA datasets.

## References

[1] Vecera M., Sana J., Lipina R., Smrcka M., Slaby O. (2018). Long Non-Coding RNAs in Gliomas: From Molecular Pathology to Diagnostic Biomarkers and Therapeutic Targets. Int. J. Mol. Sci..

[2] Wen P.Y., Kesari S. (2008). Malignant gliomas in adults. N. Engl. J. Med..

[3] Hanif F., Muzaffar K., Perveen K., Malhi S.M., Simjee S.U. (2017). Glioblastoma Multiforme: A Review of its Epidemiology and Pathogenesis through Clinical Presentation and Treatment. Asian Pac. J. Cancer Prev. APJCP.

[4] Stupp R., Mason W.P., van den Bent M.J., Weller M., Fisher B., Taphoorn M.J., Belanger K., Brandes A.A., Marosi C., Bogdahn U. (2005). Radiotherapy plus concomitant and adjuvant temozolomide for glioblastoma. N. Engl. J. Med..

[5] Zhong J., Paul A., Kellie S.J., O’Neill G.M. (2010). Mesenchymal migration as a therapeutic target in glioblastoma. J. Oncol..

[6] Ho V.K., Reijneveld J.C., Enting R.H., Bienfait H.P., Robe P., Baumert B.G., Visser O. (2014). Dutch Society for, N.-O. Changing incidence and improved survival of gliomas. Eur. J. Cancer.

[7] Tykocki T., Eltayeb M. (2018). Ten-year survival in glioblastoma. A systematic review. J. Clin. Neurosci. Off. J. Neurosurg. Soc. Australas..

[8] Ohgaki H., Dessen P., Jourde B., Horstmann S., Nishikawa T., Di Patre P.L., Burkhard C., Schuler D., Probst-Hensch N.M., Maiorka P.C. (2004). Genetic pathways to glioblastoma: A population-based study. Cancer Res..

[9] Olar A., Aldape K.D. (2014). Using the molecular classification of glioblastoma to inform personalized treatment. J. Pathol..

[10] Cancer Genome Atlas Research Network (2008). Comprehensive genomic characterization defines human glioblastoma genes and core pathways. Nature.

[11] Verhaak R.G., Hoadley K.A., Purdom E., Wang V., Qi Y., Wilkerson M.D., Miller C.R., Ding L., Golub T., Mesirov J.P. (2010). Integrated genomic analysis identifies clinically relevant subtypes of glioblastoma characterized by abnormalities in PDGFRA, IDH1, EGFR, and NF1. Cancer Cell.

[12] Wrensch M., Fisher J.L., Schwartzbaum J.A., Bondy M., Berger M., Aldape K.D. (2005). The molecular epidemiology of gliomas in adults. Neurosurg. Focus.

[13] Nicolaidis S. (2015). Biomarkers of glioblastoma multiforme. Metab. Clin. Exp..

[14] Gan H.K., Kaye A.H., Luwor R.B. (2009). The EGFRvIII variant in glioblastoma multiforme. J. Clin. Neurosci. Off. J. Neurosurg. Soc. Australas..

[15] Gourlay J., Morokoff A.P., Luwor R.B., Zhu H.J., Kaye A.H., Stylli S.S. (2017). The emergent role of exosomes in glioma. J. Clin. Neurosci. Off. J. Neurosurg. Soc. Australas..

[16] Skog J., Wurdinger T., van Rijn S., Meijer D.H., Gainche L., Sena-Esteves M., Curry W.T., Carter B.S., Krichevsky A.M., Breakefield X.O. (2008). Glioblastoma microvesicles transport RNA and proteins that promote tumour growth and provide diagnostic biomarkers. Nat. Cell Biol..

[17] Tamura K., Aoyagi M., Wakimoto H., Ando N., Nariai T., Yamamoto M., Ohno K. (2010). Accumulation of CD133-positive glioma cells after high-dose irradiation by Gamma Knife surgery plus external beam radiation. J. Neurosurg..

[18] Li L., Chakraborty S., Yang C.R., Hatanpaa K.J., Cipher D.J., Puliyappadamba V.T., Rehman A., Jiwani A.J., Mickey B., Madden C. (2014). An EGFR wild type-EGFRvIII-HB-EGF feed-forward loop regulates the activation of EGFRvIII. Oncogene.

[19] Andre-Gregoire G., Gavard J. (2017). Spitting out the demons: Extracellular vesicles in glioblastoma. Cell Adhes. Migr..

[20] Kessler T., Sahm F., Sadik A., Stichel D., Hertenstein A., Reifenberger G., Zacher A., Sabel M., Tabatabai G., Steinbach J. (2018). Molecular differences in IDH wildtype glioblastoma according to MGMT promoter methylation. Neuro-Oncology.

[21] Westphal M., Lamszus K. (2015). Circulating biomarkers for gliomas. Nat. Rev. Neurol..

[22] Cairncross G., Berkey B., Shaw E., Jenkins R., Scheithauer B., Brachman D., Buckner J., Fink K., Souhami L., Laperierre N. (2006). Phase III trial of chemotherapy plus radiotherapy compared with radiotherapy alone for pure and mixed anaplastic oligodendroglioma: Intergroup Radiation Therapy Oncology Group Trial 9402. J. Clin. Neurosci. Off. J. Neurosurg. Soc. Australas..

[23] van den Bent M.J., Carpentier A.F., Brandes A.A., Sanson M., Taphoorn M.J., Bernsen H.J., Frenay M., Tijssen C.C., Grisold W., Sipos L. (2006). Adjuvant procarbazine, lomustine, and vincristine improves progression-free survival but not overall survival in newly diagnosed anaplastic oligodendrogliomas and oligoastrocytomas: A randomized European Organisation for Research and Treatment of Cancer phase III trial. J. Clin. Oncol. Off. J. Am. Soc. Clin. Oncol..

[24] Suntsova M., Gaifullin N., Allina D., Reshetun A., Li X., Mendeleeva L., Surin V., Sergeeva A., Spirin P., Prassolov V. (2019). Atlas of RNA sequencing profiles for normal human tissues. Sci. Data.

[25] The Cancer Genome Atlas. https://portal.gdc.cancer.gov/repository.

[26] Chinese Glioma Genome Atlas. http://cgga.org.cn.

[27] Zhao Y., Wong L., Goh W.W.B. (2020). How to do quantile normalization correctly for gene expression data analyses. Sci. Rep..

[28] Borisov N., Sorokin M., Garazha A., Buzdin A. (2020). Quantitation of Molecular Pathway Activation Using RNA Sequencing Data. Methods Mol. Biol..

[29] Sorokin M., Borisov N., Kuzmin D., Gudkov A., Zolotovskaia M., Garazha A., Buzdin A. (2021). Algorithmic Annotation of Functional Roles for Components of 3,044 Human Molecular Pathways. Front. Genet..

[30] Therneau T.M. (2021). Survival Analysis [R Package Survival Version 3.2-11].

[31] (2021). Drawing Survival Curves using “ggplot2” [R Package Survminer Version 0.4.9].

[32] Dusa A. (2021). Draw Venn Diagrams [R Package Venn Version 1.10].

[33] Sorokin M., Ignatev K., Barbara V., Vladimirova U., Muraveva A., Suntsova M., Gaifullin N., Vorotnikov I., Kamashev D., Bondarenko A. (2020). Molecular Pathway Activation Markers Are Associated with Efficacy of Trastuzumab Therapy in Metastatic HER2-Positive Breast Cancer Better than Individual Gene Expression Levels. Biochemistry.

[34] Blighe K., Rana S., Lewis M. EnhancedVolcano: Publication-Ready Volcano Plots with Enhanced Colouring and Labeling. https://bioconductor.org/packages/release/bioc/vignettes/EnhancedVol-cano/inst/doc/EnhancedVolcano.html#references.

[35] GitHub Repository. https://github.com/raevskymichail/CRNDE_glioblastoma/tree/v0.1.

[36] Buzdin A., Sorokin M., Garazha A., Sekacheva M., Kim E., Zhukov N., Wang Y., Li X., Kar S., Hartmann C. (2018). Molecular pathway activation—New type of biomarkers for tumor morphology and personalized selection of target drugs. Semin. Cancer Biol..

[37] George B., Seals S., Aban I. (2014). Survival analysis and regression models. J. Nucl. Cardiol..

[38] Singh R., Mukhopadhyay K. (2011). Survival analysis in clinical trials: Basics and must know areas. Perspect. Clin. Res..

[39] Spruance S.L., Reid J.E., Grace M., Samore M. (2004). Hazard ratio in clinical trials. Antimicrob. Agents Chemother..

[40] Guan Q., Chen R., Yan H., Cai H., Guo Y., Li M., Li X., Tong M., Ao L., Li H. (2016). Differential expression analysis for individual cancer samples based on robust within-sample relative gene expression orderings across multiple profiling platforms. Oncotarget.

[41] Lu Y., Sha H., Sun X., Zhang Y., Wu Y., Zhang J., Zhang H., Wu J., Feng J. (2020). CRNDE: An oncogenic long non-coding RNA in cancers. Cancer Cell Int..

[42] Zhang J., Yin M., Peng G., Zhao Y. (2018). CRNDE: An important oncogenic long non-coding RNA in human cancers. Cell Prolif..

[43] Zheng J., Li X.D., Wang P., Liu X.B., Xue Y.X., Hu Y., Li Z., Li Z.Q., Wang Z.H., Liu Y.H. (2015). CRNDE affects the malignant biological characteristics of human glioma stem cells by negatively regulating miR-186. Oncotarget.

[44] Gao J., Chen Q., Zhao Y., Hou R. (2020). lncRNA CRNDE is Upregulated in Glioblastoma Multiforme and Facilitates Cancer Progression Through Targeting miR-337-3p and ELMOD2 Axis. OncoTargets Ther..

[45] Young M.D., Wakefield M.J., Smyth G.K., Oshlack A. (2010). Gene ontology analysis for RNA-seq: Accounting for selection bias. Genome Biol..

[46] Benjamini Y., Drai D., Elmer G., Kafkafi N., Golani I. (2001). Controlling the false discovery rate in behavior genetics research. Behav. Brain Res..

[47] Graham L.D., Pedersen S.K., Brown G.S., Ho T., Kassir Z., Moynihan A.T., Vizgoft E.K., Dunne R., Pimlott L., Young G.P. (2011). Colorectal Neoplasia Differentially Expressed (CRNDE), a Novel Gene with Elevated Expression in Colorectal Adenomas and Adenocarcinomas. Genes Cancer.

[48] Ellis B.C., Molloy P.L., Graham L.D. (2012). CRNDE: A Long Non-Coding RNA Involved in CanceR, Neurobiology, and DEvelopment. Front. Genet..

[49] Liang Q., Guan G., Li X., Wei C., Wu J., Cheng P., Wu A., Cheng W. (2020). Profiling pro-neural to mesenchymal transition identifies a lncRNA signature in glioma. J. Transl. Med..

[50] Katsushima K., Jallo G., Eberhart C.G., Perera R.J. (2021). Long non-coding RNAs in brain tumors. NAR Cancer.

[51] Moorcraft S.Y., Gonzalez D., Walker B.A. (2015). Understanding next generation sequencing in oncology: A guide for oncologists. Crit. Rev. Oncol. Hematol..

[52] Moore L.D., Le T., Fan G. (2013). DNA methylation and its basic function. Neuropsychopharmacology.

[53] Derrien T., Johnson R., Bussotti G., Tanzer A., Djebali S., Tilgner H., Guernec G., Martin D., Merkel A., Knowles D.G. (2012). The GENCODE v7 catalog of human long noncoding RNAs: Analysis of their gene structure, evolution, and expression. Genome Res..

[54] Gao Y.F., Wang Z.B., Zhu T., Mao C.X., Mao X.Y., Li L., Yin J.Y., Zhou H.H., Liu Z.Q. (2016). A critical overview of long non-coding RNA in glioma etiology 2016: An update. Tumour Biol. J. Int. Soc. Oncodev. Biol. Med..

[55] Hardwick S.A., Bassett S.D., Kaczorowski D., Blackburn J., Barton K., Bartonicek N., Carswell S.L., Tilgner H.U., Loy C., Halliday G. (2019). Targeted, High-Resolution RNA Sequencing of Non-coding Genomic Regions Associated with Neuropsychiatric Functions. Front. Genet..

[56] Rusconi F., Battaglioli E., Venturin M. (2020). Psychiatric Disorders and lncRNAs: A Synaptic Match. Int. J. Mol. Sci..

[57] Zeng T., Li L., Zhou Y., Gao L. (2018). Exploring Long Noncoding RNAs in Glioblastoma: Regulatory Mechanisms and Clinical Potentials. Int. J. Genom..

[58] Kiang K.M., Zhang X.Q., Leung G.K. (2015). Long Non-Coding RNAs: The Key Players in Glioma Pathogenesis. Cancers.

[59] Li R., Qian J., Wang Y.Y., Zhang J.X., You Y.P. (2014). Long noncoding RNA profiles reveal three molecular subtypes in glioma. CNS Neurosci. Ther..

[60] Zhang X.Q., Sun S., Lam K.F., Kiang K.M., Pu J.K., Ho A.S., Lui W.M., Fung C.F., Wong T.S., Leung G.K. (2013). A long non-coding RNA signature in glioblastoma multiforme predicts survival. Neurobiol. Dis..

[61] Han L., Zhang K., Shi Z., Zhang J., Zhu J., Zhu S., Zhang A., Jia Z., Wang G., Yu S. (2012). LncRNA pro fi le of glioblastoma reveals the potential role of lncRNAs in contributing to glioblastoma pathogenesis. Int. J. Oncol..

[62] Kraus T.F., Greiner A., Guibourt V., Lisec K., Kretzschmar H.A. (2015). Identification of Stably Expressed lncRNAs as Valid Endogenous Controls for Profiling of Human Glioma. J. Cancer.

[63] Chen Y., Wu J.J., Lin X.B., Bao Y., Chen Z.H., Zhang C.R., Cai Z., Zhou J.Y., Ding M.H., Wu X.J. (2015). Differential lncRNA expression profiles in recurrent gliomas compared with primary gliomas identified by microarray analysis. Int. J. Clin. Exp. Med..

[64] Chen W., Xu X.K., Li J.L., Kong K.K., Li H., Chen C., He J., Wang F., Li P., Ge X.S. (2017). MALAT1 is a prognostic factor in glioblastoma multiforme and induces chemoresistance to temozolomide through suppressing miR-203 and promoting thymidylate synthase expression. Oncotarget.

[65] Li H., Yuan X., Yan D., Li D., Guan F., Dong Y., Wang H., Liu X., Yang B. (2017). Long Non-Coding RNA MALAT1 Decreases the Sensitivity of Resistant Glioblastoma Cell Lines to Temozolomide. Cell Physiol. Biochem..

[66] Cai T., Liu Y., Xiao J. (2018). Long noncoding RNA MALAT1 knockdown reverses chemoresistance to temozolomide via promoting microRNA-101 in glioblastoma. Cancer Med..

[67] Jia L., Tian Y., Chen Y., Zhang G. (2018). The silencing of LncRNA-H19 decreases chemoresistance of human glioma cells to temozolomide by suppressing epithelial-mesenchymal transition via the Wnt/beta-Catenin pathway. OncoTargets Ther..

[68] Jiang P., Wang P., Sun X., Yuan Z., Zhan R., Ma X., Li W. (2016). Knockdown of long noncoding RNA H19 sensitizes human glioma cells to temozolomide therapy. OncoTargets Ther..

[69] Barry G. (2014). Integrating the roles of long and small non-coding RNA in brain function and disease. Mol. Psychiatry.

[70] Zhang X., Sun S., Pu J.K., Tsang A.C., Lee D., Man V.O., Lui W.M., Wong S.T., Leung G.K. (2012). Long non-coding RNA expression profiles predict clinical phenotypes in glioma. Neurobiol. Dis..

[71] Kiang K.M.Y., Leung G.K.K. (2017). Clinical significance of CRNDE transcript variants in glioblastoma multiforme. Noncoding RNA Res..

[72] Kiang K.M., Zhang X.Q., Zhang G.P., Li N., Cheng S.Y., Poon M.W., Pu J.K., Lui W.M., Leung G.K. (2017). CRNDE Expression Positively Correlates with EGFR Activation and Modulates Glioma Cell Growth. Target. Oncol..

[73] Jing S.Y., Lu Y.Y., Yang J.K., Deng W.Y., Zhou Q., Jiao B.H. (2016). Expression of long non-coding RNA CRNDE in glioma and its correlation with tumor progression and patient survival. Eur. Rev. Med. Pharmacol. Sci..

[74] Zheng J., Liu X., Wang P., Xue Y., Ma J., Qu C., Liu Y. (2016). CRNDE Promotes Malignant Progression of Glioma by Attenuating miR-384/PIWIL4/STAT3 Axis. Mol. Ther. J. Am. Soc. Gene Ther..

[75] Li D.X., Fei X.R., Dong Y.F., Cheng C.D., Yang Y., Deng X.F., Huang H.L., Niu W.X., Zhou C.X., Xia C.Y. (2017). The long non-coding RNA CRNDE acts as a ceRNA and promotes glioma malignancy by preventing miR-136-5p-mediated downregulation of Bcl-2 and Wnt2. Oncotarget.

[76] Wang Y., Wang Y., Li J., Zhang Y., Yin H., Han B. (2015). CRNDE, a long-noncoding RNA, promotes glioma cell growth and invasion through mTOR signaling. Cancer Lett..

[77] Li H., Li Q., Guo T., He W., Dong C., Wang Y. (2017). LncRNA CRNDE triggers inflammation through the TLR3-NF-kappaB-Cytokine signaling pathway. Tumour Biol. J. Int. Soc. Oncodev. Biol. Med..

